# Cardiac Energetics Before, During, and After Anthracycline-Based Chemotherapy in Breast Cancer Patients Using ^31^P Magnetic Resonance Spectroscopy: A Pilot Study

**DOI:** 10.3389/fcvm.2021.653648

**Published:** 2021-04-06

**Authors:** Gillian Macnaught, Olga Oikonomidou, Christopher T. Rodgers, William Clarke, Annette Cooper, Heather McVicars, Larry Hayward, Saeed Mirsadraee, Scott Semple, Martin A. Denvir

**Affiliations:** ^1^Edinburgh Imaging Facility, Queen's Medical Research Institute, University of Edinburgh, Edinburgh, United Kingdom; ^2^Centre for Cardiovascular Sciences, Queen's Medical Research Institute, University of Edinburgh, Edinburgh, United Kingdom; ^3^Edinburgh Cancer Research Centre, University of Edinburgh, Edinburgh, United Kingdom; ^4^Edinburgh Cancer Centre, Western General Hospital, Edinburgh, United Kingdom; ^5^Department of Clinical Neurosciences, Wolfson Brain Imaging Centre, University of Cambridge, Cambridge, United Kingdom; ^6^Division of Cardiovascular Medicine, Radcliffe Department of Medicine, University of Oxford Centre for Clinical Magnetic Resonance Research (OCMR), Level 0, John Radcliffe Hospital, Oxford, United Kingdom; ^7^Department of Cardiology, Royal Brompton Hospital, London, United Kingdom

**Keywords:** chemotherapy, breast cancer, cardiac energetics, troponin, ejection fraction

## Abstract

**Purpose:** To explore the utility of phosphorus magnetic resonance spectroscopy (^31^P MRS) in identifying anthracycline-induced cardiac toxicity in patients with breast cancer.

**Methods:** Twenty patients with newly diagnosed breast cancer receiving anthracycline-based chemotherapy had cardiac magnetic resonance assessment of left ventricular ejection fraction (LVEF) and ^31^P MRS to determine myocardial Phosphocreatine/Adenosine Triphosphate Ratio (PCr/ATP) at three time points: pre-, mid-, and end-chemotherapy. Plasma high sensitivity cardiac troponin-I (cTn-I) tests and electrocardiograms were also performed at these same time points.

**Results:** Phosphocreatine/Adenosine Triphosphate did not change significantly between pre- and mid-chemo (2.16 ± 0.46 vs. 2.00 ± 0.56, *p* = 0.80) and pre- and end-chemo (2.16 ± 0.46 vs. 2.17 ± 0.86, *p* = 0.99). Mean LVEF reduced significantly by 5.1% between pre- and end-chemo (61.4 ± 4.4 vs. 56.3 ± 8.1 %, *p* = 0.02). Change in PCr/ATP ratios from pre- to end-chemo correlated inversely with changes in LVEF over the same period (*r* = −0.65, *p* = 0.006). Plasma cTn-I increased progressively during chemotherapy from pre- to mid-chemo (1.35 ± 0.81 to 4.40 ± 2.64 ng/L; *p* = 0.01) and from mid- to end-chemo (4.40 ± 2.64 to 18.33 ± 13.23 ng/L; *p* = 0.001).

**Conclusions:** In this small cohort pilot study, we did not observe a clear change in mean PCr/ATP values during chemotherapy despite evidence of increased plasma cardiac biomarkers and reduced LVEF. Future similar studies should be adequately powered to take account of patient drop-out and variable changes in PCr/ATP and could include T1 and T2 mapping.

## Introduction

Anthracyclines are widely used in the treatment of breast cancer and are well-recognized to carry increased risk of cardiotoxicity (1–3). This can occur as an early, acute manifestation, or many years after treatment as late onset cardiomyopathy (4, 5). It is increasingly apparent that there may be a chronic subclinical phase associated with low grade cardiac injury with no apparent clinical impact on the contractile function of the heart (6). This period may remain latent for many years with the patient remaining asymptomatic and with apparently normal cardiac function. Identification of early and subtle cardiac dysfunction during this period could provide a window of opportunity for therapeutic intervention if those at risk or those experiencing this low-grade decline could be accurately identified. Recent studies have suggested that it might be possible to detect subclinical cardiac dysfunction using echocardiographic strain measurements of the left ventricle and that such changes might predict future risk of developing cardiac dysfunction (7, 8). Other studies have suggested that plasma levels of cardiac troponin could also provide a useful marker of early and ongoing cardiac injury following anthracycline treatment (9, 10).

Left Ventricular Ejection Fraction (LVEF), assessed by a variety of imaging methods, is recognized as insensitive for the detection of early anthracycline-induced cardiomyopathy (11). The widely used values for a clinically relevant decline in ejection fraction associated with clinical symptoms of heart failure, or an asymptomatic decrease in LVEF of 10% to <55% (10, 12) represent a relatively late manifestation of cardiac toxicity. In some cases, by the time a reduction in LVEF occurs the patient may have irreversible cardiac damage. As cancer survival rates increase there is a clear need not only to identify early markers of anthracycline myocardial toxicity but also to explore new techniques that could potentially identify baseline characteristics, pre-chemotherapy, that might predict the likelihood of developing subsequent cardiotoxicity. An accurate method of identifying, and possibly even predicting, early cardiac toxicity could aid oncologists in optimizing chemotherapy treatment whilst limiting the short and long-term cardiotoxic effects of these agents.

This study explores the potential utility of phosphorus magnetic resonance spectroscopy (^31^P-MRS) acquired using a clinical 3T MR system to identify early anthracycline-induced cardiac toxicity and baseline predisposition to cardiac injury. This technique measures the phosphocreatine/adenosine triphosphate concentration ratio (PCr/ATP), which reflects cardiac cellular energetics. The normal range of PCr/ATP is approximately 1.8–2.2, whereas decreased PCr/ATP is associated with a drop in available energy reserve in the heart, associated with many forms of heart failure (13, 14).

Conventional cardiac investigations of electrocardiogram and ultra-high sensitivity plasma cardiac troponin-I (cTn-I) have also been applied. To our knowledge PCr/ATP has not previously been used sequentially to assess cardiac injury in breast cancer patients before, during, and after chemotherapy.

## Methods

### Ethics Statement

This study was approved by the South East Scotland Research Ethics Committee (14/SS/1041) and all participants gave full written informed consent.

### Study Population and Recruitment

Twenty female breast cancer patients with no prior exposure to anthracyclines scheduled to receive anthracycline-based chemotherapy either in the adjuvant or the neoadjuvant setting were recruited from the Breast clinic at the South East Scotland Cancer Center over a period of 18 months. The combination chemotherapy regimens were either six cycles of adjuvant FEC-80 [Fluorouracil, Epirubicin (80 mg/m^3^), cyclophosphamide], or three cycles of FEC [Fluorouracil, Epirubicin (100 mg/m^3^), cyclophosphamide] followed by three cycles of Docetaxel (FEC-T). Four patients had a history of hypertension: two treated with ramipril and two with bendroflumethiazide (Table 1). None of the patients had coronary heart disease, diabetes, hypercholesterolemia, or heart failure at baseline.

**Table 1:** Patient population clinical characteristics and treatment regimens.

**Patient**	**Age range (years)**	**Smoking history**	**BMI**	**Menopausal status**	**Hyper-tension**	**Chemo-therapy**	**Trastuzumab**	**Cancer side**	**Radiotherapy**
1	60–70	No	28	Post	No	FEC-T	No	R	Yes
2	60–70	No	31	Post	No	FEC	No	R	Yes
3	40–50	No	24	Pre	No	FEC-T	No	R	No
4	50–60	No	31	Post	No	FEC80	No	L	Yes
5	40–50	Yes	28	Pre	No	FEC-T	No	R	Yes
6	50–60	No	38	Post	No	FEC-T	No	L	Yes
7	30–40	No	24	Pre	No	FEC-T	No	L	No
8	50–60	Yes	32	Post	No	FEC-T	No	L	Yes
9	60–70	No	26	Post	Yes^*^	FEC80	No	R	Yes
10	50–60	No	25	Pre	No	FEC-T	No	R	Yes
11	30–40	No	28	Pre	No	FEC80	No	L	Yes
12	50–60	No	30	Pre	No	FEC-T	No	R	Yes
13	50–60	Yes	24	Post	No	FEC-T	Yes	L	Yes
14	40–50	No	36	Pre	No	FEC-T	No	R	Yes
15	50–60	No	35	Post	Yes^*^	FEC-T	No	L	Yes
16	40–50	No	23	Pre	Yes^**^	FEC80	No	R	Yes
17	50–60	No	27	Post	Yes^**^	FEC80	No	L	Yes
18	40–50	No	26	Pre	No	FEC-T	Yes	L+R	Yes
19	30–40	Yes	30	Pre	No	FEC-T	No	L	Yes
20	50–60	No	32	Pre	No	FEC-T	No	L	Yes

*Cardiac history of any of angina, hypertension, previous myocardial infarction, heart failure. Chemotherapy regimen: FEC80—six cycles of Fluorouracil, Epirubicin (80 mg/m^3^), cyclophosphamide. FEC-T—three cycles of Fluorouracil, Epirubicin, (100 mg/m^3^), cyclophosphamide followed by three cycles of docetaxel*.

* *Hypertension treated with ramipril*.

** *Hypertension treated with Bendroflumethiazide*.

The mean time between each scan was 7 weeks (range 6–12 weeks) and was designed flexibly to allow for the oncology team to defer or postpone the next chemo-treatment for clinical reasons including chemotherapy-related side effects including nausea, fatigue, and general malaise.

Thirteen healthy volunteers (48.8 ± 11.2 years, range 31–60) with no background medical conditions and on no medications underwent a single MRI imaging protocol and a single ^31^P MRS as described for patients. Phosphocreatine/Adenosine Triphosphate Ratios and left ventricular ejection fraction were calculated for each volunteer. Scanning was repeated for three of these normal subjects to assess repeatability of measurements. The maximum difference between repeated PCr/ATP measurements and repeated LVEF measurements for volunteers defined the cut-off above which a real change could be assumed to occur in patients between pre- to mid-chemotherapy, mid- to end-chemotherapy, and pre- to end-chemotherapy. Volunteers did not undergo ECG or plasma troponin measurements and did not undergo sequential repeat MR scans.

### High Sensitivity Cardiac Troponin-I (cTn-I) Measurements

Blood samples were taken immediately prior to the first and fourth chemotherapy cycles and within 2 weeks of completion of the sixth chemotherapy cycle. Plasma Cardiac Tn-I was measured using a high sensitivity assay (ARCHITECT *STAT* Troponin I assay; Abbott Laboratories). This assay has a limit of detection of 1.2 ng/L, with a coefficient of variation of 23% at the limit of detection (1.2 ng/L) and <10% at 6 ng/L (15). The upper reference limit, determined by the manufacturer as the 99^th^ centile of samples from 4,590 healthy individuals, is 16 ng/L in women (15). As part of clinical follow-up, cTn-I was also measured within 3 months of completion of chemotherapy in patients where values increased above the normal range (0–16 ng/l) at any time point during chemotherapy.

### Cardiac Magnetic Resonance Imaging (CMRI)

Participants attended the Edinburgh Imaging QMRI facility at three time points: pre-, mid-, and end-chemotherapy. Mid-chemotherapy was defined as between cycles 3 and 4 and end-chemotherapy was within 27 ± 19 days of completing cycle 6. On each occasion participants were positioned head first supine in a 3T Verio MR scanner (Siemens Healthineers, Erlangen, Germany) between anterior and posterior parts of an 8-element cardiac ^31^P receive array coil (Rapid Biomedical, Rimpar, Germany).

### Phosphorus Magnetic Resonance Spectroscopy

**Figure 1:**
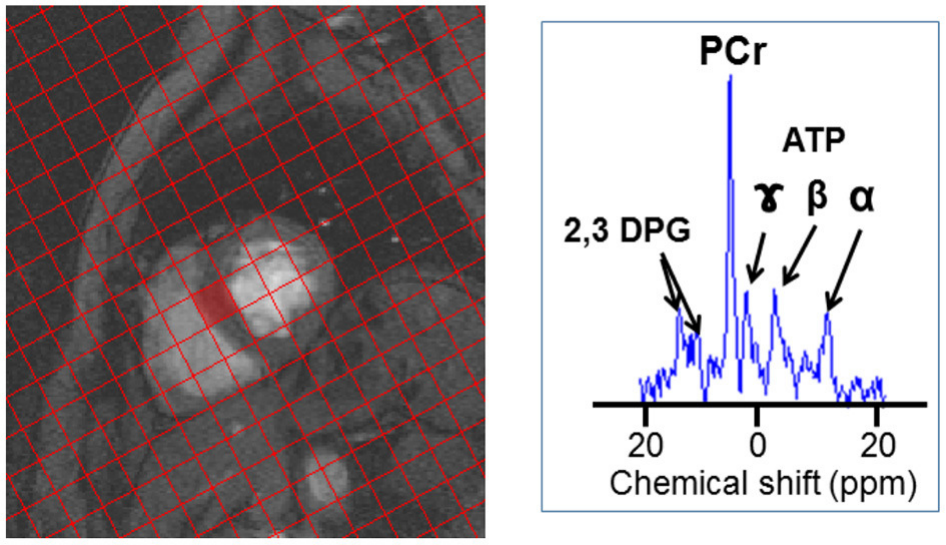
^31^P-MRS spectrum acquisition. An example of the voxel positioning for ^31^P-MRS acquisition spectrum, the spectrum acquired at the mid-septum, denoted by the red square, is analyzed. An example of a ^31^P MR spectrum is also shown (B) with resonances corresponding to phosphocreatine (PCr), γ, β, and α adenosine triphosphate (ATP), and 2,3-diphosphoglycerate (2,3 DPG) labeled.

The ^31^P MRS protocol used for this study has been described in detail elsewhere (16). In brief the total MR protocol acquisition time including positioning, set-up and acquisition of cine MR imaging to calculate ejection fraction and MRS was 60 min. ^31^P MR spectra were acquired over 30 min using a 3D UTE-CSI pulse sequence (TR/TE = 1,000/~0.6 ms, FOV = 350 × 350 × 350 mm^3^, 22 × 22 × 10 CSI matrix). A flip angle (α) of 30° was applied to the mid-septum of the myocardium. Voxels were carefully planned so that one full voxel was aligned with the mid-septum. Figure 1 shows an example of the planning of the voxel positioning. While spectra are acquired from each voxel, that acquired from the mid-septum voxel (denoted by the red square in Figure 1) is the spectrum used for analysis. The ^31^P MRS acquisition was not ECG gated.

Analysis of the ^31^P spectra was carried out using a custom Matlab implementation of AMARES (17) and was analyzed by two independent observers blinded to the time-point of analysis. This analysis quantifies the amount of phosphocreatine (PCr) and adenosine triphosphate (ATP) present in the cardiac spectrum as a concentration ratio (“PCr/ATP”). The PCr/ATP ratio was corrected for saturation effects and blood contamination. Phosphocreatine/Adenosine Triphosphate Ratios were calculated for each of the three time-points.

### Left Ventricular Ejection Fraction

A series of Cardiac Magnetic Resonance (CMR) cine images were acquired from base to apex in the short axis plane using the system's integrated body coil (TrueFISP sequence: TR/TE = 85.8/1.45 ms, α = 50°, FOV = 400 × 338 mm^2^, matrix = 256 × 205, Grappa acceleration factor = 3, slice thickness = 8 mm). Two independent operators subsequently calculated the LVEF from these images using QMass® software (Medis, Leiden, The Netherlands). This was carried out for images acquired at each time point.

We used the definition of the Cardiac Review and Evaluation Committee (CREC) supervising Trastuzumab trials which defined cardiotoxicity as a decrease in LVEF of 5 points to <55% *with* accompanying signs or symptoms of heart failure, or a decline of 10 points to <55% *without* heart failure signs or symptoms (10, 12).

### Electrocardiograms

A 12-lead ECG was acquired at each of the three time points immediately prior to each MRI scan. These were reported by a Cardiologist and any abnormalities grouped by ST segment change, left ventricular hypertrophy and changes in the QT interval.

### Statistical Analysis

All statistical analysis was carried out using Minitab 17 Statistical Software (Minitab 17 Statistical Software (2010), State College, PA: Minitab, Inc.). Data were analyzed using paired Student's *t*-tests or one-way ANOVA as appropriate. Associations between various parameters were assessed using Pearson's Coefficient. Significance was accepted at *p* ≤ 0.05.

The mean PCr/ATP ratio for age-matched female healthy controls was 1.75 ± 0.59 (range 1.11–2.18, *n* = 13). In three healthy volunteers with repeat measurements of PCr/ATP the difference in values between scans were 0.58, 0.53, and 0.20. This was consistent with other published work suggesting that a change in PCr/ATP of 0.5, with 95% confidence, could be detected with a sample size of nine patients using a similar protocol as that used in patients with heart failure (18). However, since we could not accurately predict the change in PCr/ATP associated with the anthracycline regimes used in our patients we recognized that, taking drop-out into account, we might not be adequately powered to detect a smaller change in PCr/ATP even with the proposed 20 patients agreed with our ethical review board.

## Results

### Patient Characteristics

All participants were women with median age 51 (range 31–67 years) and median body mass index 28 (range 23–36). Six patients (30%) received FEC80, fourteen (70%) received FEC-T chemotherapy, and eighteen (90%) received radiotherapy. Two patients treated with the FEC-T received Trastuzumab from the mid-chemo time point. Ten patients had left sided tumors and one had left and right sided tumors. Nine participants were post-menopausal.

All participants attended their pre-chemotherapy MRI scan while five were unable to attend the mid-chemotherapy scan due to inter-current illness and one was unable to attend the final end-chemotherapy MRI scan.

### Cardiac Magnetic Resonance Imaging (CMR)

The mean LVEF for healthy volunteers was 63.6 ± 4.5% with range 57.6–74.3%. Differences in repeated measurements of LVEF for three volunteers were 0.5, 0.6, and −0.5%, indicating a very high degree of repeatability.

Left ventricular ejection fraction was calculated for all 20 participants prior to chemotherapy, 15 at mid-chemotherapy, and 19 at end-chemotherapy.

**Figure 2:**
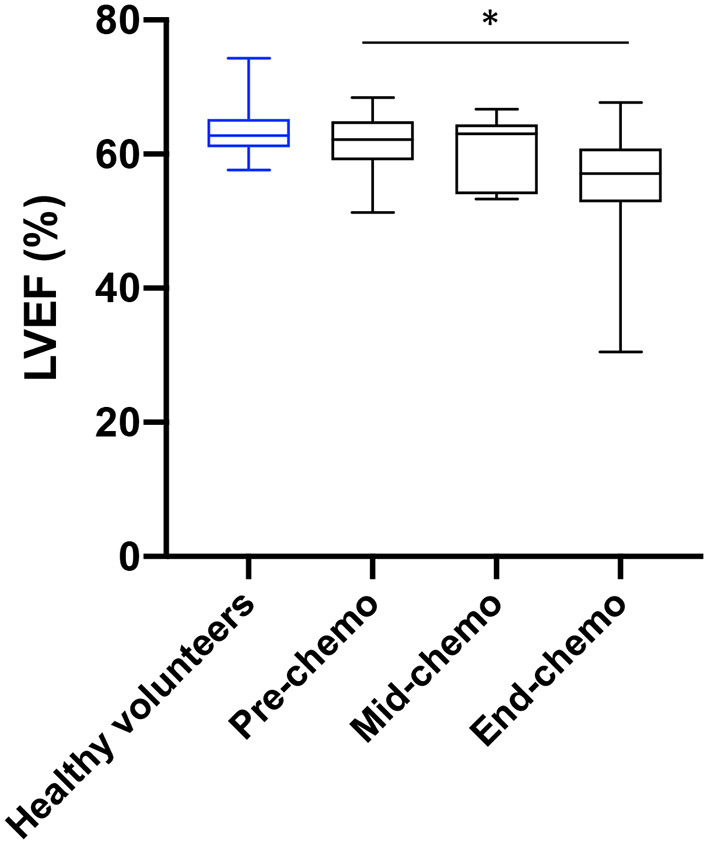
Left ventricular ejection fraction in healthy volunteers and in breast cancer patients during chemotherapy. Healthy volunteer left ventricular ejection fraction (LVEF, blue, *n* = 13) and sequential LVEF in patients (black) pre- (*n* = 20), mid- (*n* = 15), and end-chemotherapy (*n* = 19) acquired by cardiac magnetic resonance (CMR) imaging [box and whiskers plot, values are mean ± SD, and 95% confidence intervals, *P = 0.02 for change from pre-to end chemo in patients (paired *t*-test)].

The mean (± standard deviation) LVEF at pre-, mid-, and end-chemotherapy was 61.6 ± 4.4, 60.5 ± 5.2, and 56.3 ± 8.1% respectively (Figure 2). There was a significant decrease in LVEF between pre- and end-chemotherapy (*p* = 0.02). There was no significant difference in LVEF between patients receiving FEC80 (*n* = 5) and those receiving FEC-T (*n* = 12) at mid (57.7 ± 5.6 vs. 61.9 ± 4.7%, respectively, *p* = 0.14) or end-chemo time-points (55.9 ± 3.9 vs. 56.5 ± 9.6%, respectively, *p* = 0.94). Left ventricular mass did not change significantly between pre-chemo and end-chemo (83.2 ± 11.9 vs. 82.0 ± 10.7g; *p* = 0.78).

In total, six patients experienced a significant decrease in LVEF according to agreed criteria (10). Three symptomatic patients (with breathlessness) experienced a 5% decrease in LVEF from pre- to mid-chemo. Two of these patients had no further change in LVEF from mid- to end-chemo and one had a significant increase. Three additional patients experienced a decrease of 10% in LVEF from mid- to end-chemo. One patient, receiving FEC-T, became symptomatic during treatment with associated ankle swelling and breathlessness associated with a 30% fall in LVEF between pre- and end-chemo to 30.5%. This patient responded rapidly to low dose diuretics and fluid restriction and was assessed by the cardiology team with full recovery of cardiac function within 2 weeks.

### Cardiac Energetics Assessed by ^31^P-MRS

^31^P MR spectra were successfully acquired for 19 breast cancer patients pre-chemotherapy, 11 at mid-chemotherapy, and 17 end-chemotherapy. Missing scans were due to inter-current illness or technical issues for one participant at the pre-chemotherapy scan, four mid-chemotherapy, and two end-chemotherapy. An example patient ^31^P spectrum is shown in Figure 1. There was no significant difference in the mean PCr/ATP ratio between healthy controls and patients at the pre-chemo time point (1.94 ± 0.42 vs. 2.16 ± 0.46, respectively, *p* = 0.11).

**Figure 3:**
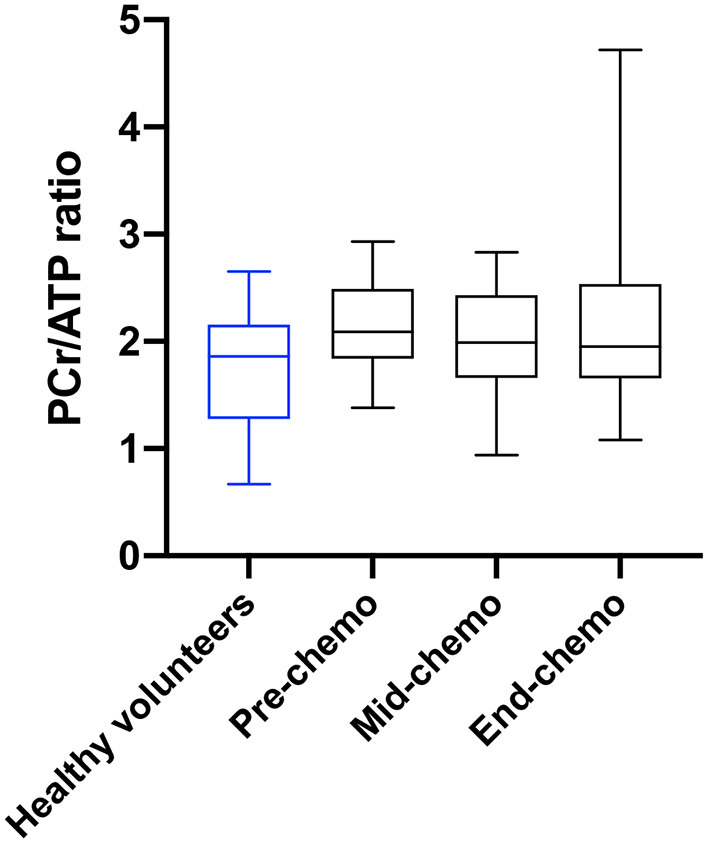
PCr/ATP ratio in healthy volunteers and in breast cancer patients during chemotherapy. Myocardial PCr/ATP ratio (box and whiskers plot) in healthy volunteers (blue) and in breast cancer patients (black) pre, mid, and at end of chemotherapy [*p* = 0.80 for pre-chemo vs. mid-chemo and *p* = 0.99 for pre to end chemo (one-way ANOVA with Tukey multiple comparison test)].

For the patient cohort there was no significant difference in mean sequential values for PCr/ATP ratio comparing pre-, mid-, and end-chemotherapy time-points. However, there was a significant negative correlation between the change in PCr/ATP from pre- to mid-chemotherapy and the subsequent change in PCr/ATP from mid- to end chemotherapy (*r* = −0.68, *p* = 0.04). The mean change in the PCr/ATP ratio at mid- and at end-chemotherapy relative to the mean baseline value is plotted in Figure 3.

### Plasma High Sensitivity Cardiac Troponin-I

**Figure 4:**
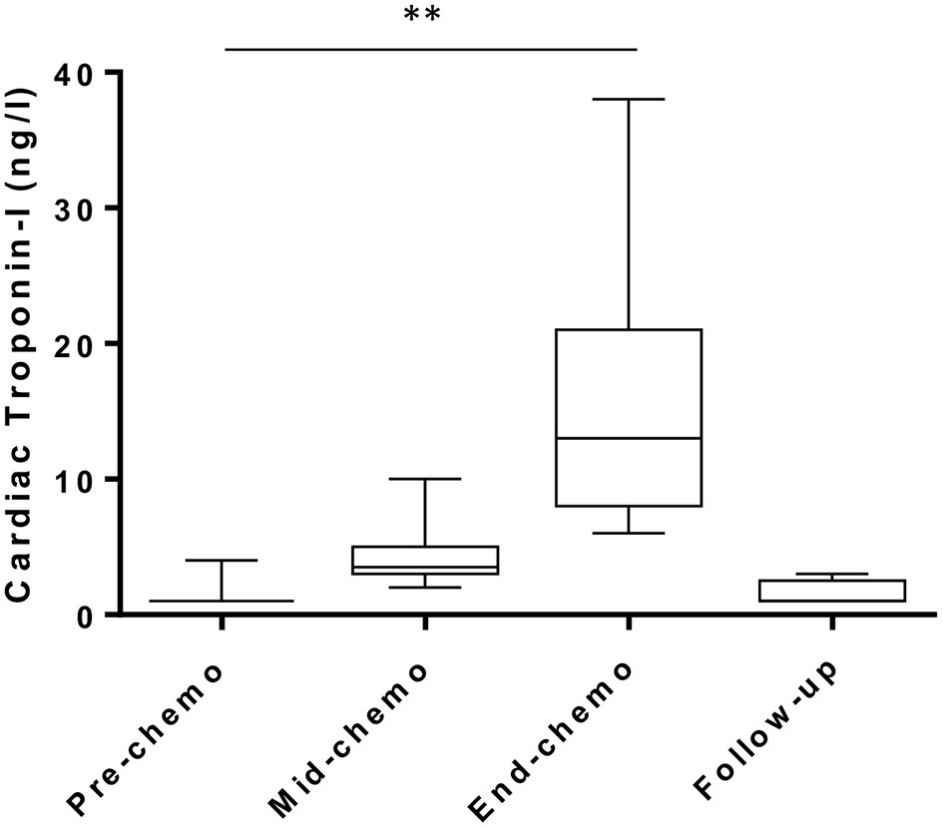
Plasma cardiac troponin levels during chemotherapy in breast cancer patients. High sensitivity cardiac troponin-I levels pre-, mid-, and end-chemotherapy [box and whiskers plot, **indicates *P* < 0.001 for change from pre-chemo (paired *t*-test)] and at 2–3 months follow-up in patients with values above the normal range at end-chemo (*n* = 9).

Plasma cardiac cTn-I-I levels (ng/L) were low-normal (<5 ng/L) in all patients at baseline prior to starting chemotherapy and increased progressively by mid-chemo with a further rise at the time of the last chemo cycle (Figure 4). The threshold cTn-I level for identifying acute coronary syndrome (ACS) in women in our center is 16 ng/L and is based on the 99^th^ upper centile of the normal range for a large sample of patients (15). In our study, while all participants showed a small but significant rise in mean cTn-I value between baseline and mid-chemo (1.35 ± 0.81 to 4.40 ± 2.64 ng/L, *p* = 0.0001) none of these values exceeded the ACS threshold (16 ng/L). By end-chemo there was a further significant increase in mean plasma cTn-I (4.40 ± 2.64 to 14.84 ± 8.73 ng/L, *p* = 0.0001) with six cTn-I values above the ACS threshold. These six patients, and a further three patients with borderline normal cTnI levels at end-chemo, were invited back 2–3 months after end of chemotherapy for longer term follow-up with repeat blood cTn-I. In all cases cTn-I had returned to normal level (mean ± SD, 1.55 ± 0.88 ng/L).

The specific type of chemotherapy regime had no impact on cTn-I levels. At mid-chemotherapy cTn-I was not significantly higher in patients receiving FEC-T compared to FEC80 (5.0 ± 2.9 vs. 3.0 ± 0.89 ng/L, respectively, *p* = 0.12) or at end-chemo in patients receiving FEC80 compared to those receiving FEC-T (18.3 ± 12.0 vs. 13.2 ± 6.7 ng/L, respectively, *p* = 0.24). As expected, the side of the cancer had no impact on cTn-I levels at any of the time points studied (end-chemo: left 15.1 ± 8.1 vs. right 15.5 ± 10.1, *p* = 0.92).

### Electrocardiograms (ECG)

There were no significant changes in PR interval or QRS duration during chemotherapy. There was a small but significant increase in the corrected QT interval (QTc) from 422 ± 15 to 438 ± 13 ms (*p* = 0.0001) with most of this increase occurring between baseline and mid-chemo. Overall, no patient showed an increase of QTc of >60 ms or a QTc above 500 ms.

### Exploratory Analysis of Possible Association Between LVEF, PCr/ATP, QT Interval, and Cardiac Troponin-*I*

A significant negative correlation was found between the change in PCr/ATP ratio and the change in LVEF from pre-chemo to end-chemo (*r* = −0.65, *p* = 0.006, Supplementary Figure). A significant negative correlation was also found between the change in PCr/ATP ratio from pre- to end-chemotherapy and the change in LVEF from mid- to end-chemotherapy (*r* = −0.77, *p* = 0.002). There was no apparent association between cTn-I and LVEF or PCr/ATP any of the time points. There was also no association found between changes in the corrected QT interval in the electrocardiogram and changes in cTn-I or changes in the QT interval and changes in LVEF. There was no apparent effect of trastuzumab treatment on cTn-I levels.

In total, six patients displayed cTn-I values above the normal range at the end-chemo timepoint. When examining each of these patients on an individual basis, no clear relationship was observed between cTn-I and baseline clinical data, including pre-existing hypertension or smoking history, LVEF or PCr/ATP ratio.

## Discussion

This study has explored the applicability of PCr/ATP ratio, measured using ^31^P MRS, to detect early anthracyclines-induced cardiac injury in a pilot cohort. The widely accepted cellular mechanism underlying anthracycline-induced cardiac toxicity is based on generation of reactive oxygen species (ROS) resulting from mitochondrial damage associated with elevated intracellular iron (19). Such damage to mitochondria is likely to cause early perturbations in high energy phosphates within cardiomyocytes and hence is the rationale for assessing ^31^P-MRS in this clinical setting. In addition, cardiotoxicity associated with breast cancer treatment is categorized as either type I, associated with irreversible cardiomyocyte death, or type II due to reversible cardiomyocyte dysfunction (9). Impaired cardiac energetics could represent a possible underlying mechanism for type II toxicity.

We compared PCr/ATP ratio with other markers of cardiotoxicity including high sensitivity plasma cardiac Tn-I, ECG parameters, and cardiac magnetic resonance imaging assessment of LVEF. While these conventional factors suggested a progressive cardiotoxic effect of chemotherapy there was no clear signal in the PCr/ATP ratio data. The possible reasons for this are discussed.

Cardioprotection during chemotherapy has been demonstrated using ACE inhibitors (20), statins (21), angiotensin receptor antagonists (22), but not metoprolol (22). Dexrazoxane has been shown to reduce cardiac troponin release during anthracycline based chemotherapy (23). Only two patients in our study received ACE inhibitors for management of high blood pressure and hence background drug therapy is unlikely to have impacted our findings. Allopurinol (24) and metformin (25) have also been shown to affect cardiac energetics and these possibly merit further study using ^31^P-MRS to assess their mechanism of action during chemotherapy.

Patient specific factors such as age and physical fitness are also known to affect PCr/ATP ratio. Jakovljevic et al. (26) found that PCr/ATP ratio was significantly reduced in older compared to younger women. They also found that older women who are physically active maintained high PCr/ATP ratios similar to younger sedentary women. Genetic factors are also known to play a part in susceptibility to cardiotoxicity in response to chemotherapy (27). Therefore, physical activity, pre-treatment levels of physical fitness, and genetics could provide intrinsic protection during chemotherapy, potentially impacting on the effects of chemotherapy on PCr/ATP.

While we observed a significant decrease in LVEF between pre and end chemotherapy by approximately 5% we also observed a negative association between changes in PCr/ATP ratio and LVEF between pre- and end-chemotherapy. This finding is not consistent with studies in dilated cardiomyopathy (unrelated to anthracyclines) where low PCr/ATP ratios were associated with reduced LVEF (28, 29) and while this was a pilot study, we had predicted a fall in PCr/ATP ratios in line with or perhaps preceding a fall in LVEF. The reasons for this are not clear. There is possibly a more complex relationship between energetics and LVEF in the setting of anthracycline chemotherapy compared to other forms of cardiomyopathy or the relatively modest reduction in mean LVEF of 5% was not sufficient to reveal a measurable change in PCr/ATP ratio during the course of the chemotherapy regimen. A further reason for the lack of clear change in PCr/ATP ratio is the possibility of a type 2 statistical error. Since the number of patients clinically well enough to have a ^31^P-MRS scan at mid-chemo time point fell from 20 to 11, this small number was at the predicted limit to allow us to detect a significant change in PCr/ATP ratio. Furthermore, this mid-chemo time point was important in that all 20 patients had been receiving anthracyclines over the previous 6 weeks in the run up to this mid-point scan. Thereafter, most patients (14) switched to docetaxel for the last three chemo-cycles thus diminishing our chances of detecting an effect on the final scan. It is therefore possible that cardiac energetics recovered in the 14 patients treated with Docetaxel for the final three chemo cycles. In addition, the relatively low cumulative dose of anthracyclines used may have limited cardiotoxicity.

Overall, the fall in LVEF, increase in QT interval on ECG and the rise in cardiac troponin together suggest that there was some degree of cardiac toxicity in our cohort. The very slight fall in PCr/ATP ratio at mid-chemo, while non-significant, is therefore highly intriguing and merits further study addressing the issues highlighted in our pilot. Furthermore, the sensitivity of ^31^P-MRS scans could be significantly improved by using higher magnetic field strengths (7T) (30, 31).

## Conclusions

This study investigated ^31^P MRS in assessing cardiac energetics of breast cancer patients undergoing chemotherapy in a pilot cohort. Our findings have highlighted the challenges of serial cardiac imaging studies during complex chemotherapy regimens and outlined a pathway by which this technique could be further explored to establish its potential for detection of cardiac energetics during chemotherapy. Future studies should take account of a potentially high level of patient drop-out, background factors that could influence cardiac PCr/ATP ratio, such as age, physical fitness, and regular medications, and should seek to improve sensitivity using 7T MRI. Future studies could include assessment of Trastuzumab on ^31^P MR myocardial imaging and changes in T1 and T2 mapping.

## Data Availability Statement

The original contributions presented in the study are included in the article/Supplementary Material, further inquiries can be directed to the corresponding author/s.

## Ethics Statement

The studies involving human participants were reviewed and approved by Lothian Research Ethics Committee. The patients/participants provided their written informed consent to participate in this study.

## Author Contributions

GM, SS, OO, and MD conceived and designed the study and wrote the ethics application. OO, HM, and LH recruited patients, collated clinical data, and collected bloods for troponin. GM, SS, CR, AC, WC, and SM performed and reported the scans. MD, OO, GM, and SS wrote the manuscript. All authors contributed to the article and approved the submitted version.

## Conflict of Interest

The authors declare that the research was conducted in the absence of any commercial or financial relationships that could be construed as a potential conflict of interest.

## Supplementary Material

Supplementary Figure Associations between the change in PCr/ATP ratio and change in LVEF during chemotherapy.
