# Supplementary figures and images for: Cardiac Energetics Before, During, and After Anthracycline-Based Chemotherapy in Breast Cancer Patients Using 31P Magnetic Resonance Spectroscopy: A Pilot Study
Source: Front Cardiovasc Med. 2021 Apr 6;8:653648. doi: 10.3389/fcvm.2021.653648 (PMC8056038; doi:10.3389/fcvm.2021.653648)

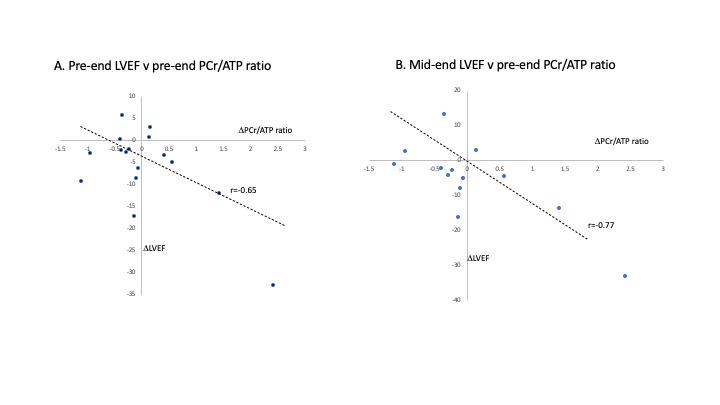

Supplement: Supplementary Figure — Associations between the change in PCr/ATP ratio and change in LVEF during chemotherapy. [file Image_1.tiff]
